# New data on the subgenus *Harpopaederus* of the genus *Paederus* (Coleoptera, Staphylinidae, Paederinae) of mainland China

**DOI:** 10.3897/zookeys.495.9484

**Published:** 2015-04-08

**Authors:** Zhong Peng, Li-Zhen Li, Mei-Jun Zhao

**Affiliations:** 1 Department of Biology, College of Life and Environmental Sciences, Shanghai Normal University, Shanghai, 200234, P. R. China

**Keywords:** Coleoptera, Staphylinidae, *Paederus*, *Harpopaederus*, new species, new records, mainland China

## Abstract

Paederus (Harpopaederus) xui Peng & Li, **sp. n.** (Sichuan: Micang Shan) is described. Additional records of five *Harpaederus* species are reported. All of these species are illustrated.

## Introduction

The widely distributed genus *Paederus* Fabricius, 1775 was previously represented in China by 39 species (Li et al. 2014; Peng et al. 2014), ten of which were placed in the subgenus *Harpopaederus* Scheerpeltz, 1957 (type species: *Paederus schoenherri* Czwalina, 1889): *Paederus antennocinctus* Willers, 2001 (Sichuan, Gansu), *Paederus gottschei* Kolbe, 1886 (Heilongjiang, Jilin; North Korea; South Korea; Russia), *Paederus konfuzius* Willers, 2001 (Shaanxi, Gansu, Sichuan), *Paederus pseudobaudii* Aleksandrov, 1934 (Heilongjiang), *Paederus apfelsinicus* Willers, 2001 (Hubei, Shaanxi), *Paederus gracilacutus* Li & Zhou, 2007 (Shaanxi, Gansu), *Paederus lineodenticulatus* Li & Zhou, 2007 (Sichuan), *Paederus dangchangensis* Li & Zhou, 2007 (Gansu), *Paederus brevior* Li, Solodovnikov & Zhou, 2014 (Shaanxi) and *Paederus multidenticulatus* Li, Solodovnikov & Zhou, 2014 (Hubei) (Li and Zhou 2007; Li et al. 2014; Smetana 2004; Willers 2001a, b).

In recent years numerous *Harpopaederus* specimens were collected during several field trips. Six species were identified, one of which is described for the first time.

## Material and methods

The material treated in this study is deposited in the following collections:

SNUC Insect Collection of Shanghai Normal University, Shanghai

cAss private collection Volker Assing, Hannover

cSch private collection Michael Schülke, Berlin

The following abbreviations are used in the text, with all measurements in millimeters:

Body length (BL) from the anterior margin of the labrum to the abdominal apex; forebody length (FL) from the anterior margin of the labrum to the posterior margin of the elytra; head length (HL) from the anterior clypeal margin to the occipital constriction; head width (HW): maximum width of head; length of antenna (AnL); length of pronotum (PL) along midline; maximum width of pronotum (PW); elytral length (EL) at the suture from the apex of the scutellum to the posterior margin of the elytra (at the sutural angles); maximum width of the elytra (EW); maximum width of abdomen (AW); length of aedeagus (AL) from the apex of the dorsal plate to the base of the aedeagal capsule.

## Results

### Paederus (Harpopaederus) apfelsinicus

Taxon classificationAnimaliaColeopteraStaphylinidae

Willers, 2001

Figs 1A , 2A, G–J

#### Material studied.

China: Hubei: 1 ♀, Shennongjia, Xiaolongtan, 05.VIII.2002, Li & Tang leg. (SNUC).

#### Comment.

**Figure 1.**
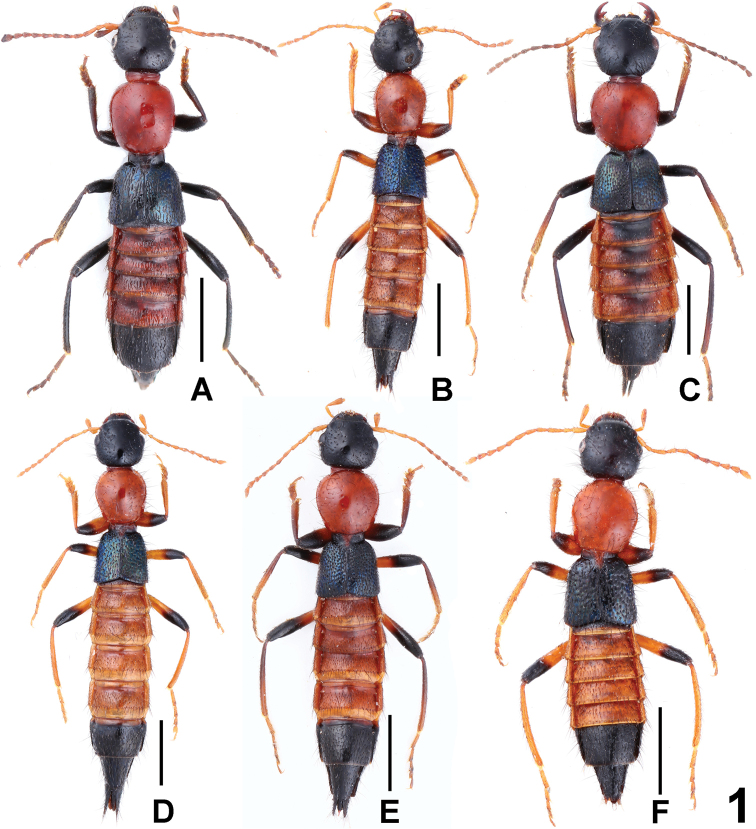
Habitus, **A** Paederus (Harpopaederus) apfelsinicus **B** Paederus (Harpopaederus) brevior **C** Paederus (Harpopaederus) gottschei **D** Paederus (Harpopaederus) gracilacutus **E** Paederus (Harpopaederus) konfuzius **F** Paederus (Harpopaederus) xui. Scale bars: 2.0 mm.

**Figure 2.**
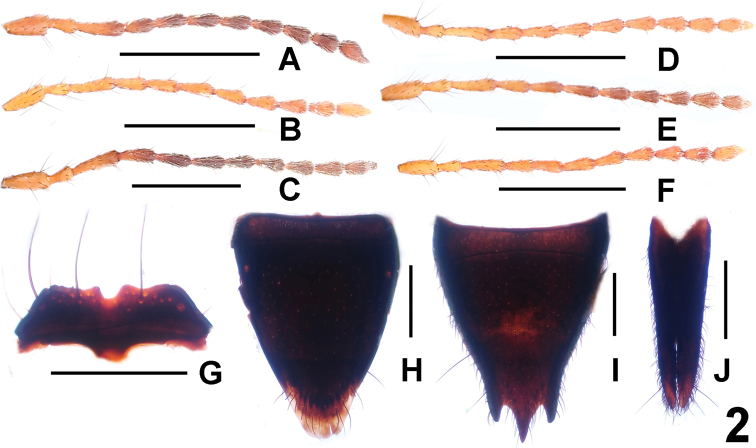
Antenna (**A**–**F**) and *Paederus apfelsinicus* (**G**–**J**), **A** *Paederus apfelsinicus* **B** *Paederus brevior* **C** *Paederus gottschei* **D** *Paederus gracilacutus* **E** *Paederus konfuzius* **F** *Paederus xui*; **G** female labrum **H** female tergite VIII **I** female sternite VIII **J** female tergite IX. Scale bars: **A**–**F** 1.0 mm; **G**–**J** 0.5 mm.

The above specimen was collected near the type locality of this species. The female external and sexual characters are illustrated in Figs 1A, 2A, 2G–J. For illustrations of the male sexual characters see Willers (2001a).

### Paederus (Harpopaederus) brevior

Taxon classificationAnimaliaColeopteraStaphylinidae

Li, Solodovnikov & Zhou, 2014

Figs 1B , 2B , 3

#### Material studied.

China: Shaanxi: 4 ♂♂, 2 ♀♀, Ningshan Hsien, Huoditang, 33°26'N, 108°26'E, 1724 m, 24–25.V.2008, Huang & Xu leg. (SNUC); 1 ♂, 1 ♀, Zhouzhi Hsien, Houzhenzi, 33°51'N, 107°50'E, 1260 m, 05.V.2008, Huang & Xu leg. (SNUC); 1 ♂, 1 ♀, Ningshan Hsien, Huoditang, 33°26'N, 108°27'E, 1500–1700 m, 12.VII.2012, Yu-Hong Pan leg. (SNUC); 7 ♂♂, 3 ♀♀, Foping Hsien, Foping Nature Reserve, 33°38'N, 107°58'E, 1250–1400 m, 18.VII.2004, Hu, Tang & Zhu leg. (SNUC).

#### Comment.

**Figure 3.**
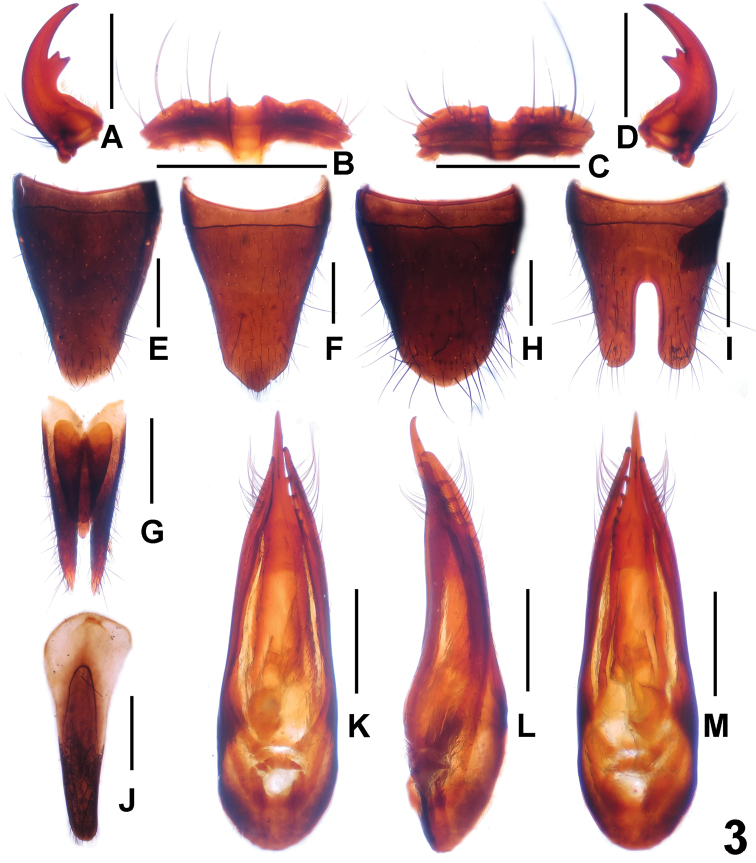
*Paederus brevior*. **A** male left mandible **B** male labrum **C** female labrum **D** male right mandible **E** female tergite VIII **F** female sternite VIII **G** female tergite IX **H** male tergite VIII **I** male sternite VIII **J** male sternite IX **K** aedeagus in ventral view **L** aedeagus in lateral view **M** aedeagus in dorsal view. Scale bars: 0.5 mm.

The original description of *Paederus brevior* is based on five type specimens from “Qinling Shan 6 km, E Xunyangba 1000–1300 m” (Li et al. 2014). The illustrations of the aedeagus provided in the original description leave no doubt that the above specimens are conspecific with the types. The external and sexual characters are illustrated in Figs 1B, 2B, 3.

### Paederus (Harpopaederus) gottschei

Taxon classificationAnimaliaColeopteraStaphylinidae

Kolbe, 1886

Figs 1C , 2C , 4

#### Material studied.

China: Jilin: 1 ♂, 2 ♀♀, Changbai Shan, 25.VII.2004, Li-Zhen Li leg. (SNUC).

#### Comment.

**Figure 4.**
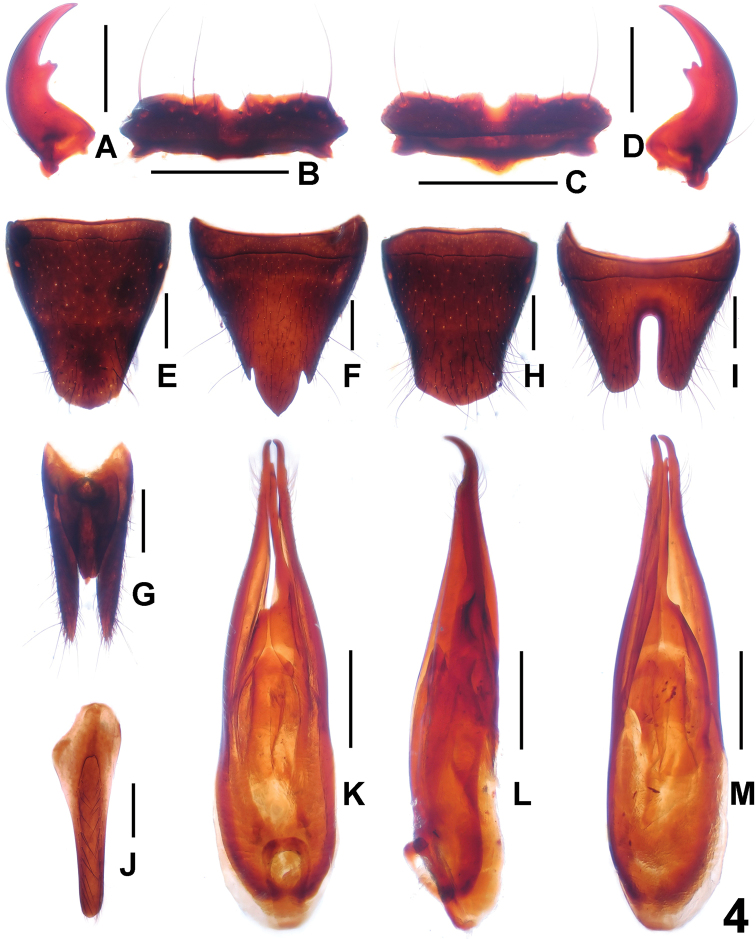
*Paederus gottschei*. **A** male left mandible **B** male labrum **C** female labrum **D** male right mandible **E** female tergite VIII **F** female sternite VIII **G** female tergite IX **H** male tergite VIII **I** male sternite VIII **J** male sternite IX **K** aedeagus in ventral view **L** aedeagus in lateral view **M** aedeagus in dorsal view. Scale bars: 0.5 mm.

The currently known distribution ranges from the Russian Far East, northern China (Heilongjiang, Jilin) to North and South Korea. The external and sexual characters are illustrated in Figs 1C, 2C, 4.

### Paederus (Harpopaederus) gracilacutus

Taxon classificationAnimaliaColeopteraStaphylinidae

Li & Zhou, 2007

Figs 1D , 2D , 5

#### Material studied.

China: Shaanxi: 5 ♂♂, 1 ♀, Nanzheng Hsien, Liping National Forest Park, 35°50'N, 106°36'E, 1400–1600 m, 15.VII.2012, Chen, Li, Ma & Zhao leg. (SNUC); 4 ♂♂, 1 ♀, Zhouzhi Hsien, Houzhenzi, Sangongli Gou, 33°51'N, 107°49'E, 1336 m, 17–19.V.2008, Huang & Xu leg. (SNUC).

#### Comment.

Although the original description is based exclusively on type specimens from Gansu, Li and Zhou (2007) repeatedly state in the text of the same article that the distribution also includes Shaanxi. The above material, whose identification is based on the illustrations provided in the original description, represents the first confirmed records from Shaanxi.

**Figure 5.**
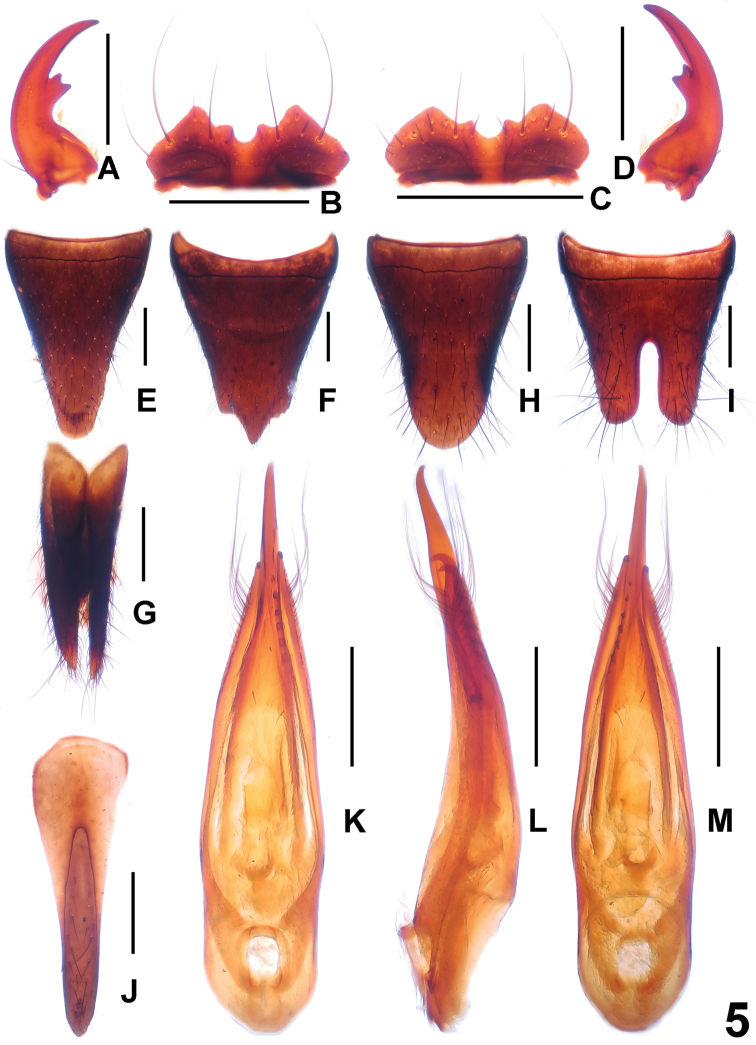
*Paederus gracilacutus*. **A** male left mandible **B** male labrum **C** female labrum **D** male right mandible **E** female tergite VIII **F** female sternite VIII **G** female tergite IX **H** male tergite VIII **I** male sternite VIII **J** male sternite IX **K** aedeagus in ventral view **L** aedeagus in lateral view **M** aedeagus in dorsal view. Scale bars: 0.5 mm.

### Paederus (Harpopaederus) konfuzius

Taxon classificationAnimaliaColeopteraStaphylinidae

Willers, 2001

Figs 1E , 2E , 6

#### Material studied.

China: Shaanxi: 6 ♂♂, Ningshaan Hsien, Pingheliang, 33°28'N, 108°29'E, 2100 m, 13.VII.2012, Chen, Li, Ma & Zhao leg. (SNUC); 1 ♂, 4 ♀♀, Zhouzhi Hsien, Daoban, 33°44'N, 107°58'E, 1900 m, 04.V.2008, Huang & Xu leg. (SNUC); 1 ♂, 1 ♀, Zhouzhi Hsien, Houzhenzi, Qinlingliangxia, 33°49'N, 107°44'E, 1820 m, 18.V.2008, Huang & Xu leg. (SNUC); 3 ♀♀, Mei Hsien, Taibai Shan, 34°01'N, 107°51'E, 1853 m, 22–23.V.2008, Huang & Xu leg. (SNUC).

#### Comment.

The original description is based on 13 type specimens from the Qinling Shan, Shaanxi (Willers 2001b). Li and Zhou (2007) recorded this species from Gansu and Sichuan, but did not specify the localities.

**Figure 6.**
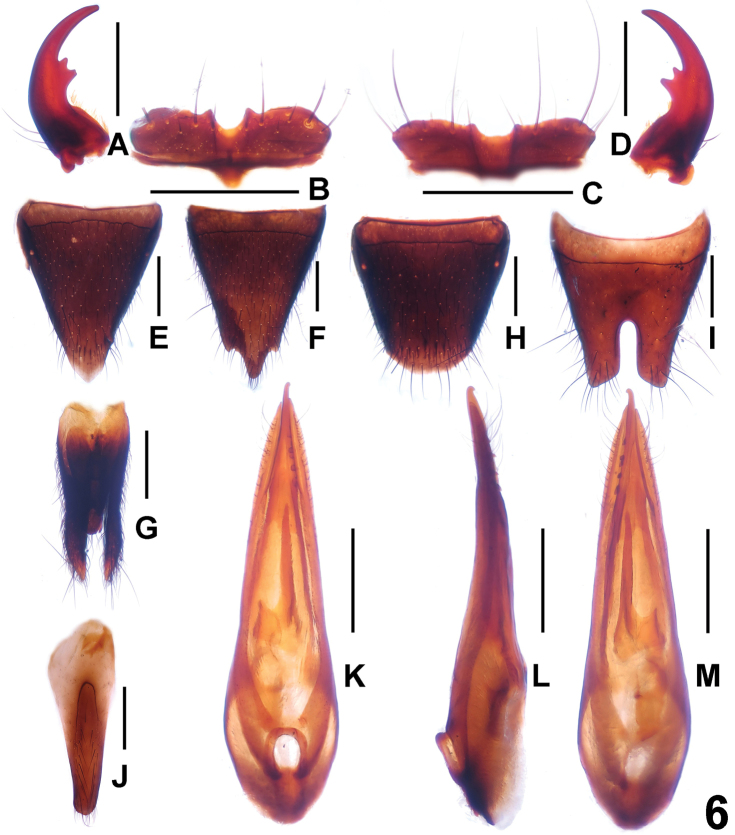
*Paederus konfuzius*. **A** male left mandible **B** male labrum **C** female labrum **D** male right mandible **E** female tergite VIII **F** female sternite VIII **G** female tergite IX **H** male tergite VIII **I** male sternite VIII **J** male sternite IX **K** aedeagus in ventral view **L** aedeagus in lateral view **M** aedeagus in dorsal view. Scale bars: 0.5 mm.

### Paederus (Harpopaederus) xui

Taxon classificationAnimaliaColeopteraStaphylinidae

Peng & Li sp. n.

http://zoobank.org/8171DF85-E235-4FA7-8671-01492B9D8DC4

Figs 1F , 2F , 7

#### Type material.

Holotype: ♂, labelled ‘China: Sichuan Province, Nanjiang Hsien, Micang Shan, Daba, 32°40‘N, 107°02‘E, 1800 m, 27.IV.2008, Huang & Xu leg.’ (SNUC). Paratypes: 2 ♂♂, 3 ♀♀, same label data as holotype (SNUC); 1♀ [teneral]: ‘CHINA (S.Shaanxi) Micang Shan, 33 km S Hanzhong, 32°44'44"N, 106°52'46"E 1360 m (stream valley, forest margin with tall herbaceous vegetation, pitfall traps, vinegar) 15.-16.VIII.2012 D.W. Wrase [30A]’ (cSch); 2♀♀: ‘CHINA: S-Shaanxi [CH12-31], Micang Shan, 40 km SW Hanzhong, 32°52'25"N, 106°37'11"E, 1530 m, N-slope, mixed secondary forest, litter and moss sifted, 16.VIII.2012, leg. M. Schülke’ (cSch, cAss).

#### Description.

Measurements (in mm) and ratios: BL: 8.67–9.34; FL: 4.72–4.95; HL: 0.93–0.98; HW: 1.07–1.11; AnL: 2.72–2.95; PL: 1.23–1.26; PW: 1.10–1.13; EL: 0.87–0.94; EW: 1.13–1.20; AW: 1.74–1.77; AL: 1.38–1.41; HL/HW: 0.84–0.87; HW/PW: 0.96–0.98; HL/PL: 0.76–0.78; PL/PW: 1.10–1.13; EL/PL: 0.71–0.75; diameter of eye: 0.34–0.36.

Habitus as in Fig. 1F. Coloration: head, labrum and apical segments black, labial palpi and antennae yellowish brown; pronotum brownish red; elytra blackish blue with metallic luster; first four abdominal segments reddish brown; legs yellowish brown, with apical halves of femora black.

Head wider than long, widest across eyes; punctation coarse and sparse; interstices glossy. Mandibles without distinct sexual dimorphism. Eyes distinctly convex. Antenna as in Fig. 2F.

Pronotum nearly oviform, strongly convex in cross-section; punctures somewhat sparser and shallower than those of head.

Elytra trapeziform; punctation coarse, defined and dense. Hind wings reduced. Metatarsomere I as long as combined length of metatarsomeres II and III.

Abdomen distinctly broader than elytra; punctation coarse; interstices with very shallow microsculpture; posterior margin of tergite VII without palisade fringe.

**Figure 7.**
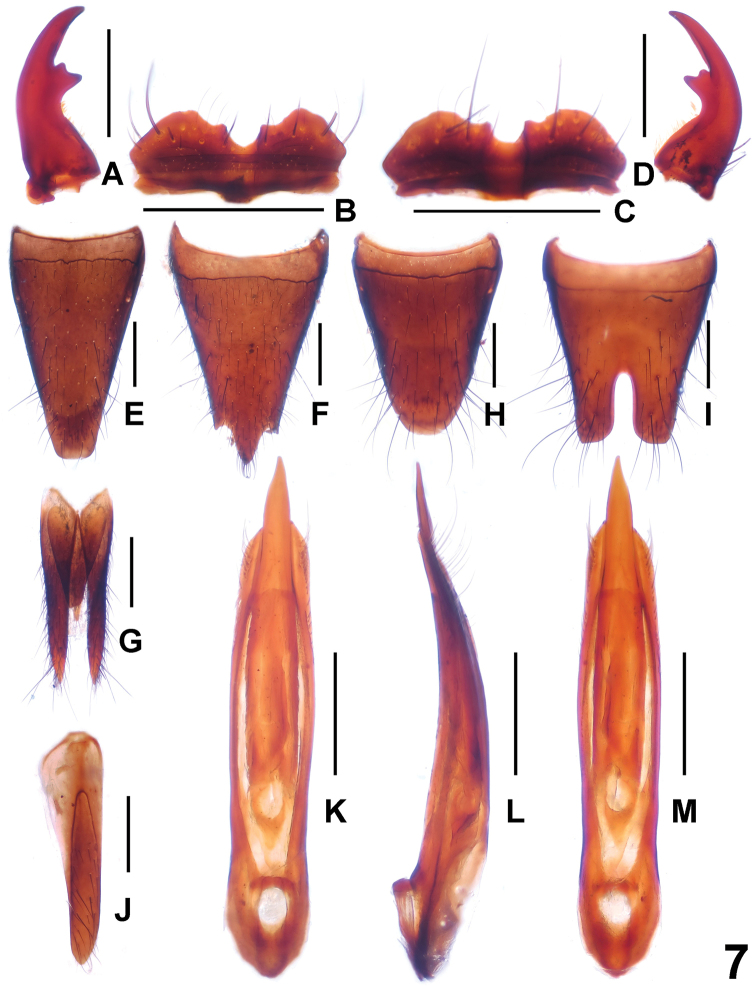
*Paederus xui*. **A** male left mandible **B** male labrum **C** female labrum **D** male right mandible **E** female tergite VIII **F** female sternite VIII **G** female tergite IX **H** male tergite VIII **I** male sternite VIII **J** male sternite IX **K** aedeagus in ventral view **L** aedeagus in lateral view **M** aedeagus in dorsal view. Scale bars: 0.5 mm.

Male. Anterior margin of labrum (Fig. 7B) distinctly sinuate; each mandible (Fig. 7A, D) with single bicuspidate tooth. Posterior margin of tergite VIII (Fig. 7H) convex; sternite VII unmodified; sternite VIII (Fig. 7I) with deep posterior incision, this incision approximately 0.4 times as long as sternite VIII; sternite IX as in Fig. 7J; aedeagus as in Fig. 7K–M; dorsal plate of median lobe nearly symmetric, long and slender; parameres very slender and curved apically; internal sac without sclerotized spines.

Female. Labrum as in Fig. 7C. Posterior margin of tergite VIII (Fig. 7E) truncate; posterior margin of sternite VIII (Fig. 7F) with narrowly triangular median process; tergites IX and X as in Fig. 7G.

#### Distribution and natural history.

The type locality is situated in the Micang Shan to the north of Nanjiang, northern Sichuan. The specimens were sifted from leaf litter in a mixed deciduous forest at an altitude of 1,800 m.

#### Etymology.

The species is dedicated to Wang Xu, who collected some of the type specimens.

#### Comparative notes.

As can be inferred from the highly similar sexual characters, the new species is allied to the geographically close *Paederus antennocinctus* Willers, 2001 (Sichuan: Jiuzhaigou; Gansu: Qiujia Dam), from which it differs by the coloration of legs, the somewhat smaller size, the shape of the labrum, the longer dorsal plate of the aedeagus and the nearly trifurcate posterior margin of the female sternite VIII.

## Supplementary Material

XML Treatment for Paederus (Harpopaederus) apfelsinicus

XML Treatment for Paederus (Harpopaederus) brevior

XML Treatment for Paederus (Harpopaederus) gottschei

XML Treatment for Paederus (Harpopaederus) gracilacutus

XML Treatment for Paederus (Harpopaederus) konfuzius

XML Treatment for Paederus (Harpopaederus) xui
